# Colchicine ultrasensitivity of peripheral-blood lymphocytes from patients with non-Hodgkin's lymphoma.

**DOI:** 10.1038/bjc.1980.102

**Published:** 1980-04

**Authors:** J. H. Scarffe, J. Prudhoe, J. V. Garrett, D. Crowther

## Abstract

Incubation for 20 h in low concentrations of colchicine has been shown to kill the peripheral blood lymphocytes (PBL) of patients with chronic lymphocytic leukaemia (CLL), whereas at least a 10,000 x higher concentration of colchicine is required to kill lymphocytes from a normal donor. This ultrasensitivity of CLL lymphocytes to low doses of colchicine was confirmed in 19/20 PBL samples, 5/6 lymph nodes, and in the one totally replaced marrow studied. PBL from 75 patients with non-Hodgkin's lymphoma (NHDL) were examined for colchicine ultrasensitive (CUS) cells similar to those found in CLL. All the patients had less than 5 x 10(9)/1 morphologically normal circulating lymphocytes. PBL from 45 healthy donors and 39 patients with diseases other than leukaemia or lymphoma were used as controls. CUS cells were detected in 24 (32%) of the 75 patients. The CUS cells were considered to represent blood involvement with malignant lymphocytes for three reasons. First, there was an association with marrow involvement (P less than 0.05) which usually accompanies involvement of the blood with morphologically abnormal cells. Secondly, 23 (77%) of the 30 involved lymph nodes, marrows and spleens studied were CUS. Thirdly, there was a close correlation with the presence of a monoclone of B lymphocytes demonstrated by surface markers (P less than 0.01).


*Br. J. Cancer* (1980) **41**, 593

### COLCHICINE ULTRASENSITIVITY OF PERIPHERAL-BLOOD LYMPHOCYTES FROM PATIENTS WITH NON-HODGKIN'S LYMPHOMA

J. H. SCARFFE*, J. PRUDHOE*, J. V. GARRETT† AND D. CROWTHER*

*From the *Cancer Research Campaign Department of Medical Oncology and †Department of Haematology, Christie Hospital, Manchester*

Received 17 July 1979 Accepted 3 January 1980

**Summary.—Incubation for 20 h in low concentrations of colchicine has been shown to kill the peripheral blood lymphocytes (PBL) of patients with chronic lymphocytic leukaemia (CLL), whereas at least a 10,000 × higher concentration of colchicine is required to kill lymphocytes from a normal donor. This ultrasensitivity of CLL lymphocytes to low doses of colchicine was confirmed in 19/20 PBL samples, 5/6 lymph nodes, and in the one totally replaced marrow studied.**

**PBL from 75 patients with non-Hodgkin's lymphoma (NHDL) were examined for colchicine ultrasensitive (CUS) cells similar to those found in CLL. All the patients had less than $5 \times 10^9$/l morphologically normal circulating lymphocytes. PBL from 45 healthy donors and 39 patients with diseases other than leukaemia or lymphoma were used as controls. CUS cells were detected in 24 (32%) of the 75 patients. The CUS cells were considered to represent blood involvement with malignant lymphocytes for three reasons. First, there was an association with marrow involvement ($P < 0.05$) which usually accompanies involvement of the blood with morphologically abnormal cells. Secondly, 23 (77%) of the 30 involved lymph nodes, marrows and spleens studied were CUS. Thirdly, there was a close correlation with the presence of a monoclone of B lymphocytes demonstrated by surface markers ($P < 0.01$).**

A PRELIMINARY COMMUNICATION by Thomson & Robinson (1967) reported that the peripheral-blood lymphocytes (PBL) of 7 patients with chronic lymphocytic leukaemia (CLL) were killed when incubated in $10^{-7}$M colchicine (0·04 μg/ml) for 20 h. Lymphocytes from healthy individuals were resistant to at least 10,000 × this concentration during the same incubation period. Further studies have confirmed this initial finding of differential sensitivity of CLL lymphocytes (Thomson *et al.*, 1972; Schrek *et al.*, 1976). Colchicine ultrasensitive (CUS) lymphocytes have also been reported in the peripheral blood of 9/23 patients with lymphoproliferative disease other than CLL (Thomson *et al.*, 1974). They studied a heterogeneous group of patients with non-Hodgkin's lymphoma (NHDL), macroglobulinaemia or unexplained lymphocytosis.

Thomson's report (1974) prompted our group in 1975 to use colchicine ultrasensitivity in addition to cell-surface markers to study early blood involvement of patients with NHDL. In a preliminary report of this work, 20/51 patients with NHDL had circulating CUS lymphocytes (Scarffe *et al.*, 1977). All the patients with CUS lymphocytes had lymphocyte counts below $7 \times 10^9$/l.

Schrek *et al.* (1978) found CUS lymphocytes in the peripheral blood of 10/13 patients with NHDL. Four of these patients, however, had lymphocyte counts greater than $7 \times 10^9$/l and two more had

Correspondence to: Dr J. H. Scarffe, Cancer Research Campaign, Department of Medical Oncology, Christie Hospital, Withington, Manchester M20, England.

circulating large morphologically abnormal cells.

There were four main aims to this study. The first was to confirm the colchicine ultrasensitivity of CLL lymphocytes. The second was to confirm the presence of CUS lymphocytes in the peripheral blood of patients with NHDL, looking specifically at those patients with normal lymphocyte morphology and count ($< 5 \times 10^9$/l). The third was to determine whether the apparently normal lymphocytes which were CUS represented a population of circulating malignant lymphoid cells, using surface-marker studies, and investigation of tissues from lymph nodes, spleens and marrows involved with NHDL. The fourth was to relate the finding of CUS cells in the peripheral blood of patients with NHDL to the Rappaport histopathological classification, clinical stage, marrow involvement and prognosis.

### PATIENTS, MATERIALS AND METHODS

*Patients.*—Peripheral-blood samples from 45 healthy donors (age range 18–65 years) and from 39 patients suffering from medical conditions other than leukaemia or lymphoma (age range 4–71 years) were used as controls. Tumour cells were obtained from involved lymph nodes, totally replaced marrow samples, or spleens removed for diagnostic or therapeutic reasons from 7 patients with chronic lymphatic leukaemia (CLL) and 31 with NHDL. Lymphocytes from 2 lymph nodes which showed reactive change only were studied. Peripheral-blood samples were obtained from 20 patients with CLL and 75 with NHDL.

Lymphnode biopsies from patients with NHDL were reviewed and classified according to Rappaport (1966). Patients were staged using the Ann Arbor classification for Hodgkin's disease (Carbone *et al.*, 1971). Staging laparotomies were not performed, but intensive staging techniques including marrow aspirate and trephines, percutaneous liver biopsies, cerebrospinal fluid examination, and abdominal lymphography were all used as indicated.

All patients with NHDL were studied before treatment. Six of the 20 patients with CLL had been treated previously. In these patients the studies were carried out when the treatment had been stopped for at least 3 months.

*Materials and methods.*—Lymphocyte preparations from lymphnode and spleen samples were obtained by gentle teasing and disaggregation with forceps and scissors. Lymphocyte preparations were assessed for viability by Trypan-blue exclusion. Preparations containing less than 90% viable cells were not tested with colchicine. Lymphocytes were separated from defibrinated peripheral blood, and heparinized marrow samples using a Ficoll–Triosil density gradient. PBL from 2 normal controls were separated further into T-rich and B-rich populations by a rosetting technique (Wybran *et al.*, 1973). The technique for testing colchicine ultrasensitivity (CUS) was similar to that described by Thomson *et al.* (1972). The lymphocytes obtained were washed and suspended in tissue-culture Medium 199 (Burroughs Wellcome) at a concentration of $1{\cdot}5 \times 10^6$/ml with 30% autologous serum. Seven 0·5ml aliquots of the suspension were incubated for 24 h in 5% $CO_2$ at 37°C. Colchicine (Koch-Light) was added at the start of the incubation period to 6 of the aliquots to give final concentrations ranging from $10^{-2}$ to $10^{-7}$ molar. The seventh aliquot acted as a control.

The number of cells killed was determined by assessing the percentage showing nuclear pyknosis in a smear wet-fixed in Susa and stained with Mayer's haemalum (Trowell, 1952, 1955). Five hundred cells were assessed in each aliquot.

Lymphocyte surface-marker studies were performed and assessed as described previously (Garrett *et al.*, 1979).

Marrow aspirates were examined for the number of lymphocytes and the presence of any immature cells. The upper limit of normal for lymphocytes was taken as 20% of the nucleated cells; 20–25% lymphocytes was considered equivocal, and 25% or over evidence of infiltration. If there were more than 5% abnormal immature lymphocytes, this was also taken as evidence of marrow involvement. The marrow trephine was considered to be involved if there was a significant diffuse, paratrabecular or focal infiltration of lymphocytes.

*Statistical analysis.*—Each patient or control studied gave a 7-point curve for cell death

(nuclear pyknosis). To reduce the complexity of comparing over 200 7-point pyknosis curves, a principal-components analysis was performed (Kendal, 1975). The first and second components represented over 90% of the information contained in each curve. A plot of the first against the second component was used to generate scattergrams on which each individual curve was represented by one point. Results from the controls and patients could then be grouped and compared. Seventy-five per cent of the information was contained in the first component, and it was clear from the scattergrams that this could be used to compare pyknosis curves. A single number could then be used to represent each curve.

### RESULTS

### *Controls and chronic lymphocytic leukaemia (CLL) patients*

Typical examples of the 7-point pyknosis curve for PBL from normal healthy donors and CLL patients are shown in Fig. 1. The normal donors showed < 10% pyknotic cells at all concentrations except $10^{-2}$M colchicine. Most CLL patients, however, had a slightly higher percentage of pyknotic cells in the aliquot incubated without colchicine than normal controls, and > 90% pyknosis at all concentrations used ($10^{-7}$–$10^{-2}$M colchicine). The marked difference in CUS between CLL cells and normal lymphocytes was clearly demonstrated.

FIG. 1.—Examples of typical 7-point % pyknosis curves of peripheral blood lymphocytes (PBL) from normal healthy donors, patients with chronic lymphatic leukaemia (CLL), and non-Hodgkin's lymphoma (NHDL).

FIG. 2.—Scattergram of the 1st and 2nd principal components of the 7-point % pyknosis curves of PBL from 45 healthy donors and 39 patients suffering from medical conditions other than leukeamia or lymphoma.

Scattergrams of the 1st and 2nd components showed no difference between the healthy normal controls and the patients with medical conditions other than CLL or lymphoma; they are therefore shown together in Fig. 2. Any figure for the first component greater than the 97·5 percentile of the results obtained from the 84 controls (*i.e.* > 0·62) was considered positive for the presence of CUS cells. The first component increases with increasing percentage of pyknotic cells at low concentrations of colchicine. In the examples shown in Fig. 1, the values for the first component of the pyknosis curves of the normal and CLL patients were − 2, and 4. Two results in the control group were greater than the 97·5 percentile limit of 0·62 for the first component, with values of 0·7 and 0·97. The first was a healthy 18-year-old technician whose test result was normal on two further occasions. The second was a boy 4 years old who developed acute lymphoblastic leukaemia 6 months later. At the time of the test he had a nonspecific illness, with only 1% blast cells in the marrow.

FIG' 3.—Scattergram of the 1st and 2nd principal components of the 7-point % pyknosis curves or PBL from 20 patients with chronic lymphatic leukaemia (CLL).

FIG. 4.—Graph of % CLL cells in a mixture of normal and CLL lymphocytes against the corrected % pyknotic cells at each proportion of the mixture, calculated from

$$\frac{P_o - P_n \cdot N}{P_{CLL}}$$

where $P_o$ = observed % pyknotic cells
$P_n$ = % pyknotic cells in normal lymphocytes
N = proportion of normal lymphocytes in mixture
$P_{CLL}$ = proportion of pyknotic cells in CLL lymphocytes

The T-enriched populations from 2 normal donors contained 91% and 95% T cells, and 7% and 4% B cells respectively. The B-enriched population contained 2% and 9% T cells, and 63% and 53% B cells respectively. There was a slight increase in CUS in the B-enriched population, but these results were well within the limits of the normal controls, with first-principal components of −2·27 and −1·35.

Of 20 PBL samples obtained from patients with CLL, 19 contained CUS lymphocytes (Fig. 3). Lymphnode biopsies were studied in 6 of these patients, and the lymphocytes were CUS in 5. Lymphocytes from one involved marrow were studied, and these were also CUS.

One patient was found to have cells resistant to colchicine in both the peripheral blood and in an involved lymph node. This patient's PBL had originally shown CUS, but as his disease progressed, with an increasing lymphocytosis, the percentage of ultrasensitive cells decreased, although the absolute number remained the same. This change in CUS was not paralleled by a change in surface markers. IgG λ surface immunoglobulin was demonstrated on 80–90% PBL at all times, and also from the lymphnode biopsy specimen.

To ascertain the accuracy of colchicine ultrasensitivity in detecting circulating abnormal lymphocytes, a mixing experiment was performed in which lymphocytes from a CLL donor were mixed in an increasing proportion with lymphocytes from a normal donor and incubated under the standard conditions with $10^{-6}$M colchicine. The percentage of pyknotic cells reflected accurately the percentage of CLL cells in the mixture after correction had been made for the number of pyknotic cells in the normal lymphocyte population, and for the resistant lymphocytes in the CLL population at each concentration of cells (Fig. 4).

*Non-Hodgkin's lymphoma patients*

CUS lymphocytes were detected in 23 (77%) of the 30 samples of histologically involved lymph nodes, marrows or spleens from patients with NHDL (Table I); the

TABLE I.—*Colchicine ultrasensitivity of lymphocytes from histologically involved lymph nodes, marrows, and spleens from patients with non-Hodgkin's lymphoma*

| Lymphnode pathology* | CUS+ | Studied |
|---|---|---|
| NLWD | 4 | 6 |
| DLWD | 6 | 6 |
| NML/H | 0 | 1 |
| DML/H | 1 | 1 |
| NLPD | 1 | 1 |
| DLPD | 10 | 13 |
| DH | 1 | 2 |
| Total | 23 (77%) | 30 |

* Histopathological classification based on Rappaport (1966).
† 1st principal component > 0·62.
NLWD = Nodular lymphocytic well differentiated.
DLWD = Diffuse lymphocytic well differentiated.
NML/H = Nodular mixed lymphocytic/histiocytic.
DML/H = Diffuse mixed lymphocytic/histiocytic.
NLPD = Nodular lymphocytic poorly differentiated.
DLPD = Diffuse lymphocytic poorly differentiated.
NH = Nodular histiocytic.
DH = Diffuse histiocytic.
DU = Diffuse undifferentiated.

lymphocytes from 2 lymph nodes which showed reactive change only were not CUS.

The percentage pyknosis curves for PBL from patients with NHDL gave 3 patterns: one similar to normal controls, one similar to that of CLL cells, and one intermediate. The example in Fig. 1 shows a lymphocyte preparation from a patient with NHDL in which 45% of the cell population was pyknotic at low concentrations of colchicine, the remainder becoming pyknotic only at $10^{-2}$M colchicine. Two populations of lymphocytes were present; one ultrasensitive to small doses of colchicine similar to CLL lymphocytes, and another similar to the normal control lymphocytes. The first principal component of the pyknosis curve was 1·2.

PBL from 75 NHDL patients were studied for CUS. All the patients had PBL counts below $5 \times 10^9$/l and the lymphocytes appeared normal by light microscopy. Twenty-four (32%) of the patients were found to have circulating CUS cells (*i.e.* 1st principal component > 0·62) (Fig. 5).

Lymphocytes from lymph nodes and peripheral blood were studied in 17/75

FIG. 5.—Scattergram of the 1st and 2nd principal components of the 7-point % pyknosis curves of PBL from 75 patients with non-Hodgkin's lymphoma (NHDL).

patients with NHDL. CUS cells were detected in lymphnode specimens from 13 patients, of whom 6 had circulating CUS cells. None of the patients whose lymphnode cells were resistant to colchicine had detectable sensitive cells in the peripheral blood. The presence of CUS cells in the blood was not related to the stage of the disease. Five of the 18 patients (28%) with localized disease (Stages I & II) and 19 of the 57 patients (33%) with generalized disease (Stages III & IV) had CUS cells detected in the peripheral blood (Table II). There was, however, a statistically significant association between the presence of CUS cells in the blood, and bone marrow infiltration with lymphoma cells (Table III): only 8 of the 43 (19%) with a normal marrow, compared with 12 out of 25 patients (48%) whose marrow was infiltrated, had CUS cells in the peripheral blood ($P < 0{\cdot}05$). Four of the 7 patients whose marrow studies were equivocal had circulating CUS cells.

Eight of 11 patients with a diffuse lymphocytic well differentiated lymphoma (DLWD) were found to have blood involvement, compared with 6/25 patients with a diffuse lymphocytic poorly differentiated lymphoma (DLPD). CUS cells in the blood were found in only 1 of 12

TABLE II.—*Colchicine ultrasensitivity of PBL from patients with non-Hodgkin's lymphoma*

| Lymphnode pathology* | Stage 1 & II | | Stage III & IV | | All stages | |
|---|---|---|---|---|---|---|
| | CUS | Studied | CUS | Studied | CUS | Studied |
| NLWD | 0 | 2 | 4 | 8 | 4 | 10 |
| DLWD | 1 | 1 | 7 | 10 | 8 | 11 |
| NML/H | – | – | 0 | 1 | 0 | 1 |
| DML/H | – | – | 2 | 2 | 2 | 2 |
| NLPD | 2 | 3 | 1 | 9 | 3 | 12 |
| DLPD | 2 | 8 | 4 | 17 | 6 | 25 |
| NH | – | – | 0 | 1 | 0 | 1 |
| DH | 0 | 4 | 1 | 8 | 1 | 12 |
| DU | – | – | 0 | 1 | 0 | 1 |
| | 5 (28%) | 18 | 19 (33%) | 57 | 24 (32%) | 75 |

TABLE III.—*Colchicine ultrasensitivity of PBL from patients with non-Hodgkin's lymphoma with and without marrow infiltration*

| Marrow status | CUS | Studied |
|---|---|---|
| Normal | 8 (19%) | 43 |
| Equivocal | 4 | 7 |
| Infiltrated | 12 (48%) | 25 |
| Total | 24 (32%) | 75 |

$\chi^2_1 = 5.24$ $P < 0.05$ for comparison of normal with infiltrated marrow.

patients whose lymphnode histology was classified as diffuse histiocytic (DH) (Table II).

There was a good correlation between the presence of circulating monoclonal B lymphocytes and CUS cells in the 23 patients with NHDL in whom both studies were performed (Table IV).

TABLE IV.—*Association of colchicine ultrasensitivity of PBL from patients with non-Hodgkin's lymphoma and the presence of a monoclone of B lymphocytes*

| | Clone +ve | Clone −ve | Total |
|---|---|---|---|
| CUS | 7 | 1 | 8 |
| not CUS | 3 | 12 | 15 |
| Total | 10 | 13 | 23 |

$\chi^2_1 = 7.12$ $P = < 0.01$.

A monoclone was present if the ratio of light chains for Ig-bearing lymphocytes was greater than $4\kappa$ to $1\lambda$ or $2\lambda$ to $1\kappa$ (Garrett *et al.*, 1979).

### *Response to chemotherapy*

The response to chemotherapy was studied in 26 patients in whom the CUS of lymphoma cells from lymph node, marrow or spleen was known. The patients were all treated with drug combinations which included vincristine. CLL cells have been shown *in vitro* to be ultrasensitive to vincristine as well as colchicine (Schrek, 1974). No difference in response was found between those patients with CUS and those without.

Five Stage I and II patients with peripheral-blood involvement (Table II) were analysed for signs of early dissemination over a follow-up of 1–3 years. All the patients are alive and well; one patient with NLPD lymphoma quickly relapsed in the marrow, the 4 other patients remain well without any sign of disease, but 3 received chemotherapy as an adjunct to radiotherapy.

### DISCUSSION

The results in this study confirm the findings of Thomson *et al.* (1972) that the circulating lymphocytes in CLL are nearly always CUS. Lymphocytes from involved lymph nodes and marrows were also found to be CUS in patients with CLL.

A mixing experiment demonstrated that CUS can be used to detect the percentage of CLL cells accurately in a mixture with normal lymphocytes.

The difference between the CUS of CLL lymphocytes which are predominantly B cells and normal cells which are predominantly T cells is not a simple preferential killing of B cells. In the T- and B-cell enrichment experiment, even when there were 50 and 60% B cells the 1st principal component of the pyknosis curve fell well within the normal range.

The presence of a high proportion of CUS cells in 77% of tissue biopsy samples from 30 patients with NHDL is a similar result to that found by Schrek *et al.* (1978). They reported that 22 (85%) of 26 lymph-node specimens showed CUS, but only 1/22 reactive nodes studied as controls. The 2 reactive lymph nodes in this study were both resistant to colchicine.

About one-third of the 75 patients with NHDL whose lymphocyte count was below $5 \times 10^9$/l were shown to have circulating CUS cells. These circulating CUS cells are likely to be malignant for three reasons. First, the CUS cells were more commonly found in patients with marrow infiltration in whom blood involvement might be expected. Marrow involvement is the usual precursor to involvement of blood with morphologically abnormal cells. Secondly, a high proportion of cells from involved lymph nodes, spleens and marrows were CUS. The third piece of evidence for the CUS cells being malignant is the close correlation with the presence of a monoclone of B lymphocytes demonstrated by surface marker studies. There were, however, 3/10 patients whose PBL contained a monoclone but whose lymphocytes were not CUS. This is, perhaps, what one would expect, since 20% of lymphoma tissue biopsy samples were not CUS. When cells from these resistant tumours migrate into the peripheral blood they may be detectable by surface marker studies but not by colchicine ultrasensitivity.

The ratio of kappa ($\kappa$) to lambda ($\lambda$) light-chain-containing surface Ig was used to determine the presence of a monoclone of B lymphocytes. In most patients the normal lymphocytes were only partially replaced by the monoclonal cells. This mixture of normal and abnormal cells made it impossible to quantitate the absolute number of monoclonal cells to compare with the number of CUS cells present, and to detect very early blood involvement. Circulating CUS cells were detected in one patient with a DLWD lymphoma, in whom a monoclone of lymphocytes was not detected (Table IV). An equal number of $\kappa$- and $\lambda$-staining cells were detected, indicating a slight increase in $\lambda$-staining cells but not in sufficient number to fulfill the criteria for a $\lambda$ monoclone of twice as many $\lambda$- as $\kappa$-staining cells. The normal lymphocytes were probably masking the presence of a monoclone, but the abnormal cells were clearly detected by CUS.

A recent study demonstrated a monoclone of circulating B lymphocytes in 25/50 patients with NHDL, using surface markers (Garrett *et al.*, 1979). The presence of a monoclone was shown to be closely associated with marrow infiltration. More patients were found to have abnormal circulating cells in the surface-marker study than in the present study for two reasons. All lymphoma cells are not CUS, and the upper limit for lymphocyte count was higher at $6 \times 10^9$/l in the surface-marker study.

Ault (1979) has confirmed this finding, using a flow cytofluorometric method to detect small numbers of unsuspected monoclonal B lymphocytes in the blood of 11/25 lymphoma patients with morphologically normal circulating lymphocytes.

Circulating CUS cells were detected in all the major histopathological groups of NHDL. They were most commonly detected in DLWD lymphomas, less often in DLPD lymphomas and rarely in DH lymphoma. This could either be because some groups are less CUS or that circulating malignant lymphocytes are less common.

In this series all 6 biopsy samples of lymphoid tissue from patients with DLWD lymphoma and 10/13 biopsy samples from patients with a DLPD lymphoma were CUS. Unfortunately only 2 lymph nodes with a DH pattern were studied, one of which was CUS. Schrek *et al.* (1978) were able to test 7 lymph nodes involved with a DH lymphoma, 5 of which contained CUS cells. Thus it appears that there were no major differences in colchicine sensitivity of tissue biopsy specimens between the different histopathological groups, and if the cells are present in the peripheral blood in sufficient numbers they should be detected by colchicine ultrasensitivity.

There was a statistically significant correlation between the presence of circulating CUS cells and marrow infiltration ($P = < 0{\cdot}05$) in the 75 patients studied. The frequency of marrow infiltration, however, varied greatly between the different histopathological groups. Infiltration was found in 7/11 patients with DLWD lymphoma, 6/25 patients with DLPD lymphoma but only 2/12 patients with DH lymphoma. Although the numbers are small they reflect the relative frequency of marrow infiltration reported in a larger series (Rosenberg, 1975). The distribution of disease appears to be different in the various histopathological groups. DLWD lymphomas have a high incidence of marrow involvement and circulating CUS cells, whereas in DH lymphomas marrow infiltration and circulating CUS cells are less common. The DLPD lymphomas fall between the two extremes. There were, however, 8/43 patients with no evidence of lymphomatous infiltration of the marrow who had CUS cells in the peripheral blood. This finding suggests the possibility that these cells were derived from involved lymphoid tissues other than the marrow. There may, however, have been a sampling error in a patchily infiltrated marrow.

The case of the child who later developed acute lymphoblastic leukaemia (ALL) is interesting, since 9/11 patients with ALL whose peripheral blood lymphoblasts have been tested showed ultrasensitivity to colchicine (Scarffe, in preparation). If circulating CUS cells can be detected in ALL patients with minimal marrow infiltration this could be used to detect early relapse.

The reason that malignant cells from patients with CLL or NHDL are ultrasensitive to colchicine is unknown. No difference has been found in the uptake of colchicine by normal and CLL lymphocytes (Thomson *et al.*, 1972). CLL cells also demonstrate ultrasensitivity to the Vinca alkaloids which have similar biological actions to colchicine, in that they will bind to microtubules and cause metaphase arrest (Schrek, 1974). A difference between the microtubules of normal and malignant lymphocytes may therefore explain the differential sensitivity to colchicine. A difference in tubulin has recently been shown using antitubulin antibodies. A "nucleus-associated tubulin-containing structure" was found in virtually all lymphocytes from normal subjects, but in a considerably lower number of CLL lymphocytes (Dighiero *et al.*, 1978). This hypothesis of the mode of action of colchicine, however, does not explain the finding that the ionophore A23187, which causes uptake into the cell of calcium and other divalent ions, induces resistance to colchicine and vincristine (Schrek *et al.*, 1978).

Further studies are required to evaluate ultrasensitivity as an aid to diagnosis and staging of NHDL patients. Preliminary studies have shown that colchicine ultrasensitivity may be useful in distinguishing between lymph nodes involved with NHDL and those which show a reactive histological pattern. It has also been suggested that colchicine ultrasensitivity may be useful in deciding whether an anaplastic tumour is of epithelial or lymphoid origin (Schrek *et al.*, 1978).

The screening of the peripheral blood of patients with NHDL for CUS cells is a simple inexpensive test to perform and the results are available within 24 h. It may be possible in patients shown to have disseminated disease by the presence of CUS cells to avoid other invasive or expensive staging procedures. The absence of CUS cells in the peripheral blood, however, does not exclude disseminated disease, since $\sim 20\%$ of lymphomas are expected to show colchicine resistance.

The authors wish to thank the members of the Manchester Lymphoma Group for permission to study their patients. They are also grateful to Mr R. Swindell for his expert statistical help, Dr O. G. Dodge and Dr J. S. Whittaker for the lymphnode histopathological diagnosis and Mrs E. N. Morgan for secretarial assistance.

### REFERENCES

AULT, K. A. (1979) Detection of small numbers of monoclonal B lymphocytes in the blood of patients with lymphoma. *N. Engl. J. Med.*, **300**, 1401.

CARBONE, P. (1971) Report of the Committee on Hodgkin's Disease Staging Classification. *Cancer Res.*, **31**, 1860.

DIGHIERO, G., KARSENTI, E., FOLLEZOU, J. & BORNENS, M. (1978) Visualization of tubulin in lymphocytes. I, Comparison of normal and chronic lymphocytic leukaemia (CLL) lymphocytes. *Blood*, **51**, 1031.

GARRETT, J. V., NEWTON, R. R. & SCARFFE, J. H. (1979) Abnormal peripheral blood lymphocytes and bone marrow infiltration in non Hodgkin's lymphoma. *Br. J. Haematol.*, **42**, 41.

KENDAL, M. (1975) Principal components Chap. 2, In *Multivariate Analysis*. London: Charles Griffin & Co. p. 13.

RAPPAPORT, H. (1966) Tumours of the haematopoietic system. In *Atlas of Tumour Pathology*. Washington: Armed Forces Institute of Pathology, Section 3, Fasc. 8. p. 13.

ROSENBERG, S. A. (1975) Bone marrow involvement in the non-Hodgkin's lymphomata. *Br. J. Cancer*, **31** (Suppl. 11), 261.

SCARFFE, H., PRUDHOE, J. & CROWTHER, D. (1977) Colchicine ultrasensitivity of principal blood lymphocytes in lymphoid malignancies. *Br. J. Cancer*, **36**, 418.

SCHREK, R. (1974) Cytotoxicity of vincristine to normal and leukaemic lymphocytes. *Am. J. Clin. Pathol.*, **62**, 1.

SCHREK, R., MESSMORE, H. L., KNOSPE, W. H. & STEFANI, S. S. (1976) A colchicine-sensitivity test for leukaemic lymphocytes. *Scand. J. Haematol.*, **16**, 357.

SCHREK, R., ZELMAR, M. & STEFANI, S. (1978) Cytology and colchicine sensitivity of viable cells from lymph nodes with malignant lymphoma. *Cancer*, **41**, 1845.

THOMSON, A. E. R. & ROBINSON, M. A. (1967) Cytocidal action of colchicine *in vitro* on lymphocytes in chronic lymphocytic leukaemia. *Lancet*, ii, 868.

THOMSON, A. E. R., O'CONNOR, T. W. E., WETHERLEY-MEIN, G. (1972) Killing and characterizing action of colchicine *in vitro* on lymphocytes of chronic lymphocytic leukaemia. *Scand. J. Haematol.*, **9**, 231.

THOMSON, A. E. R., O'CONNOR, T. W. E. & WETHERLEY-MEIN, G. (1974) Selective killing by colchicine *in vitro* of lymphocytes in chronic lymphocytic leukaemia. In: *Proceedings of Eighth Leucocyte Culture Conference*. New York: Academic Press. p. 665.

TROWELL, O. A. (1952) The culture of lymph nodes *in vitro*. *Exp. Cell Res.*, **3**, 79.

TROWELL, O. A. (1955) The culture of lymph nodes in synthetic media. *Exp. Cell Res.*, **9**, 258.

WYBRAN, J., CHANTLER, S. & FUDENBERG, H. (1973) Isolation of normal T cells in chronic lymphatic leukaemia. *Lancet*, **1**, 126.


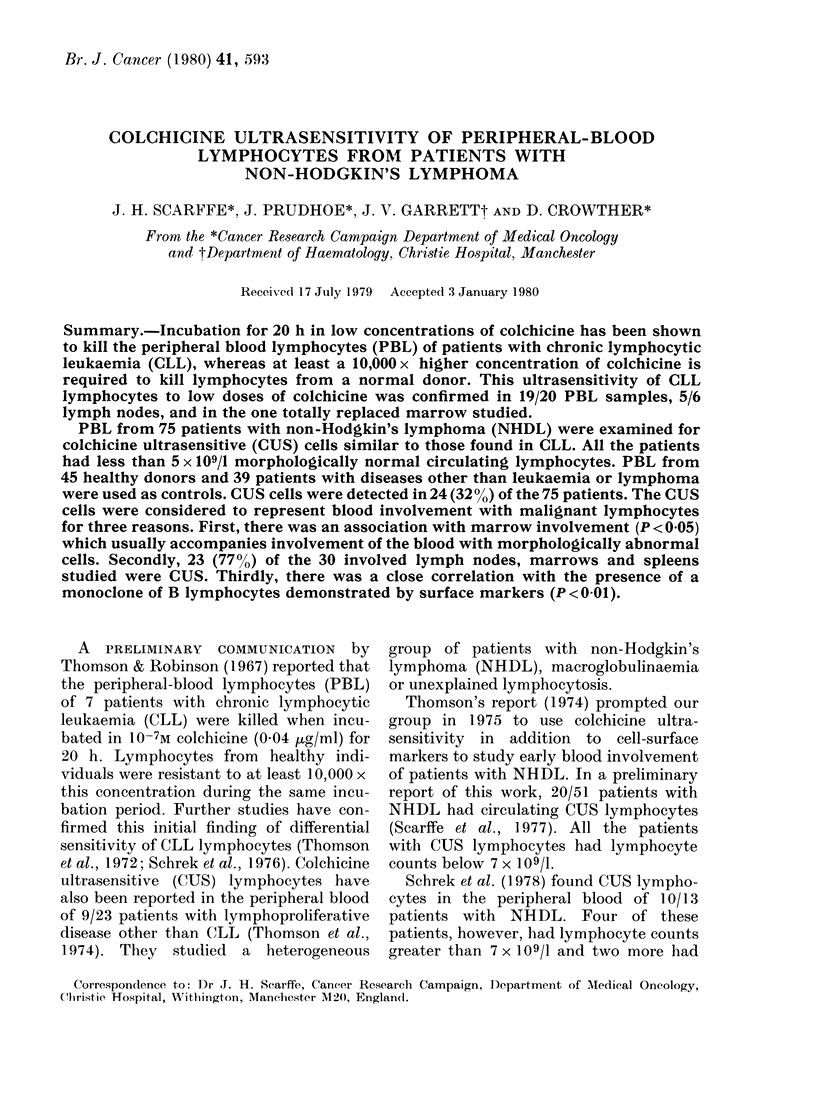

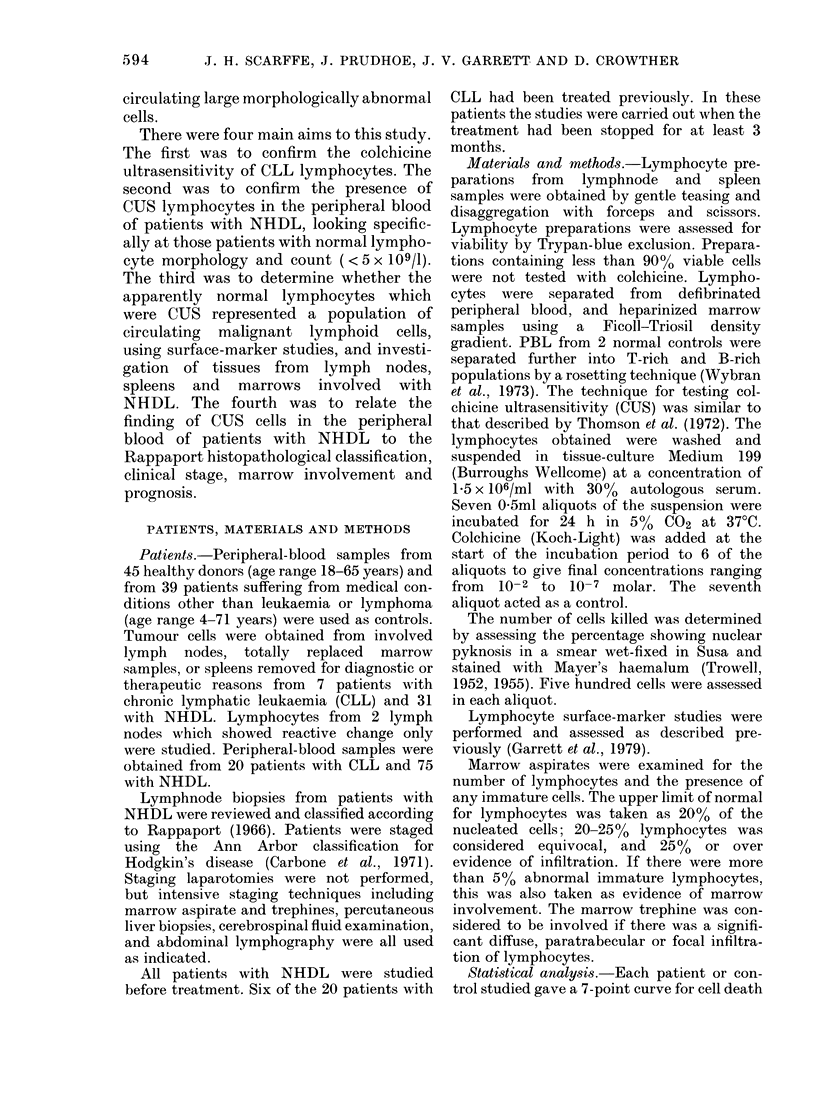

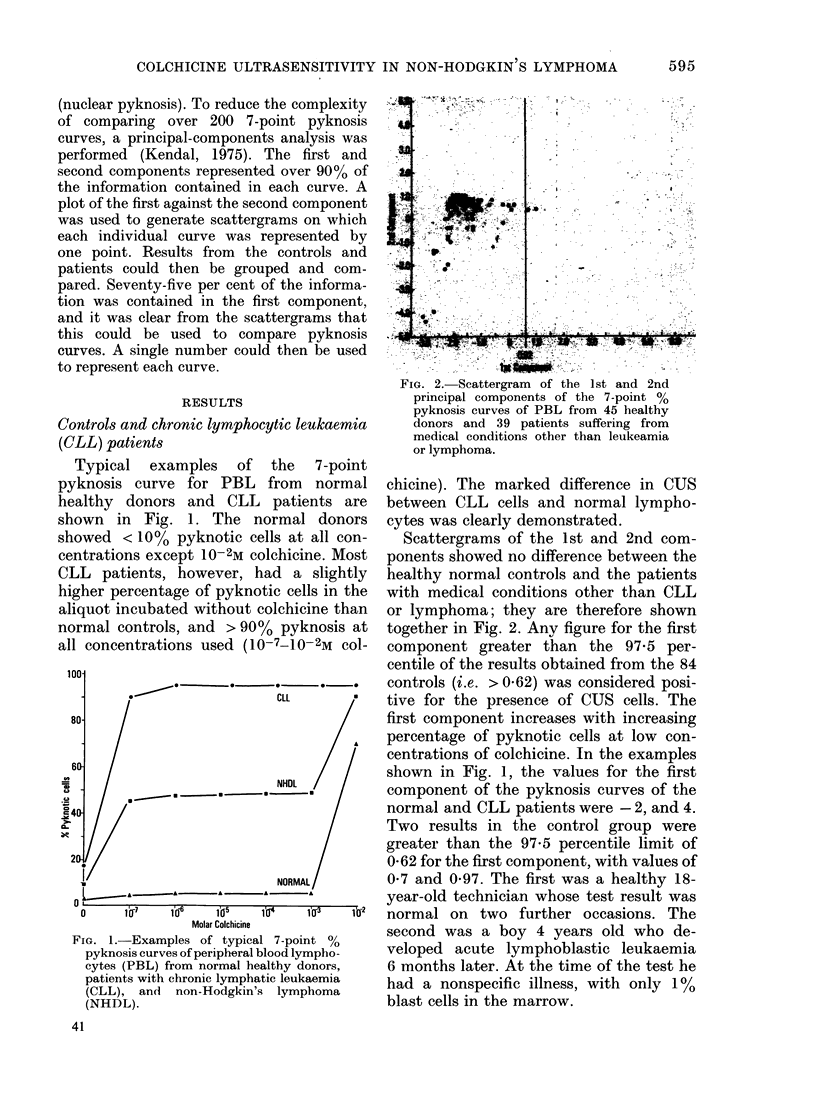

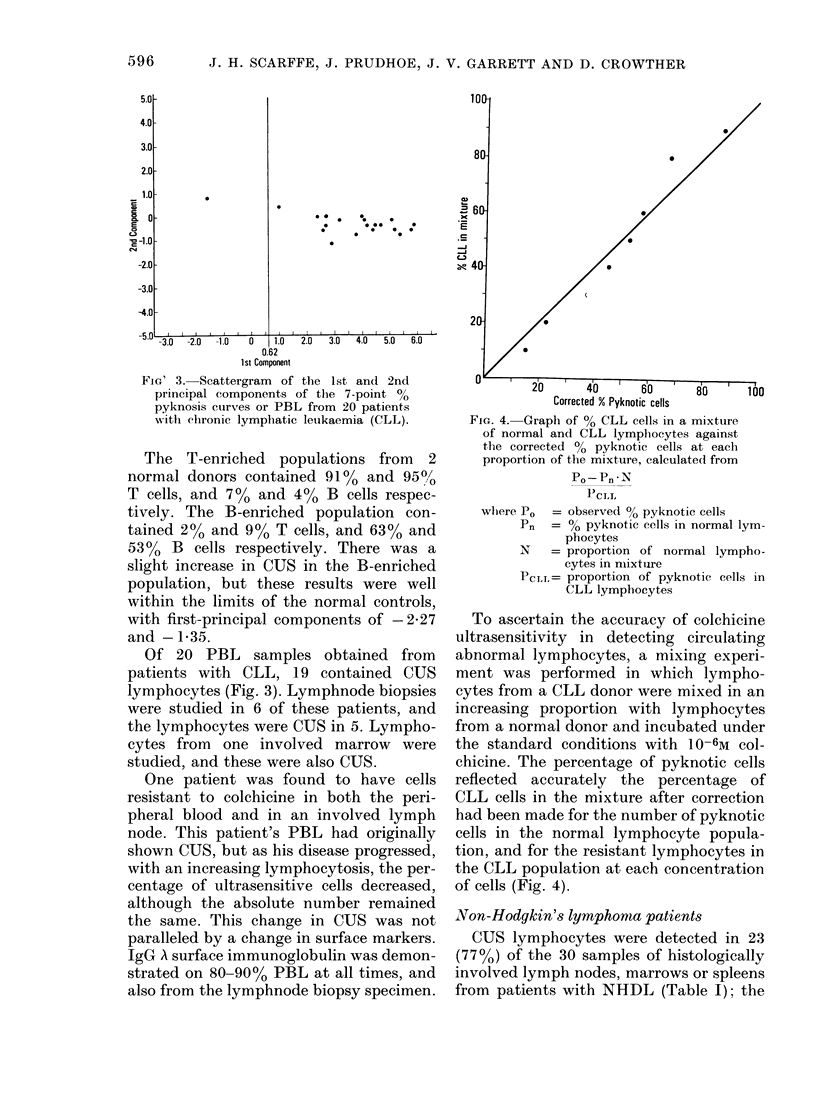

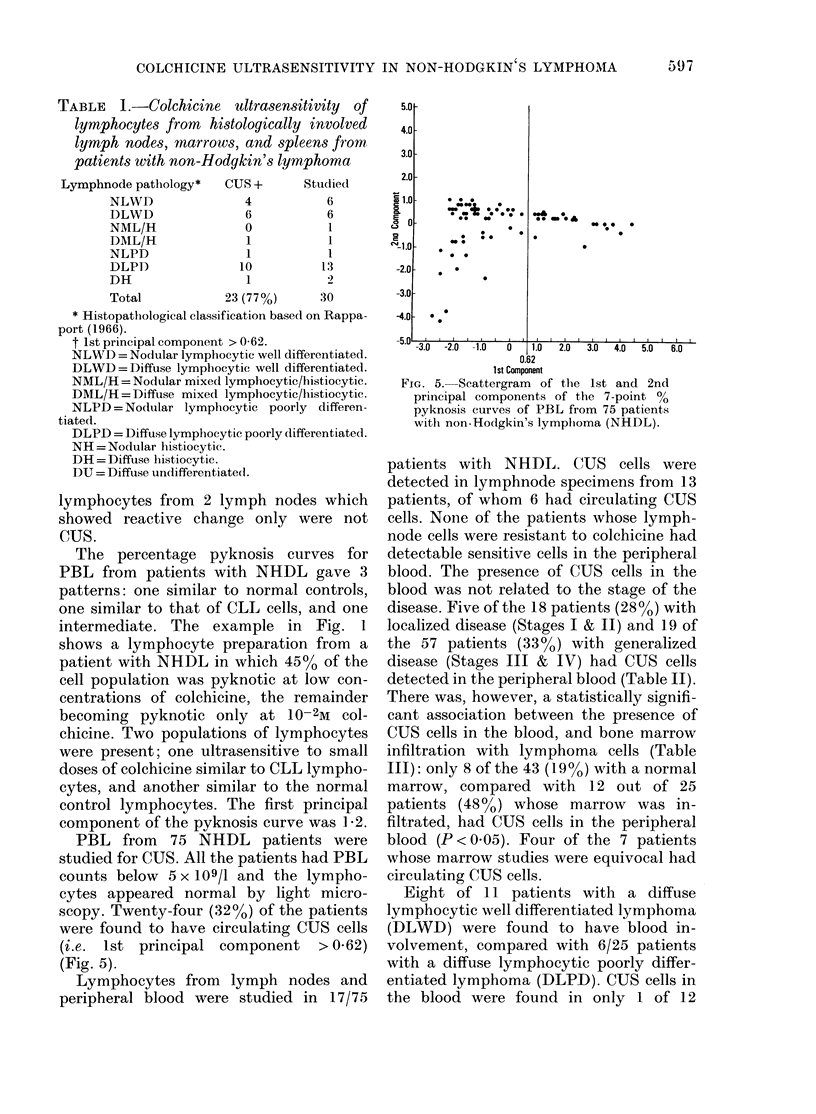

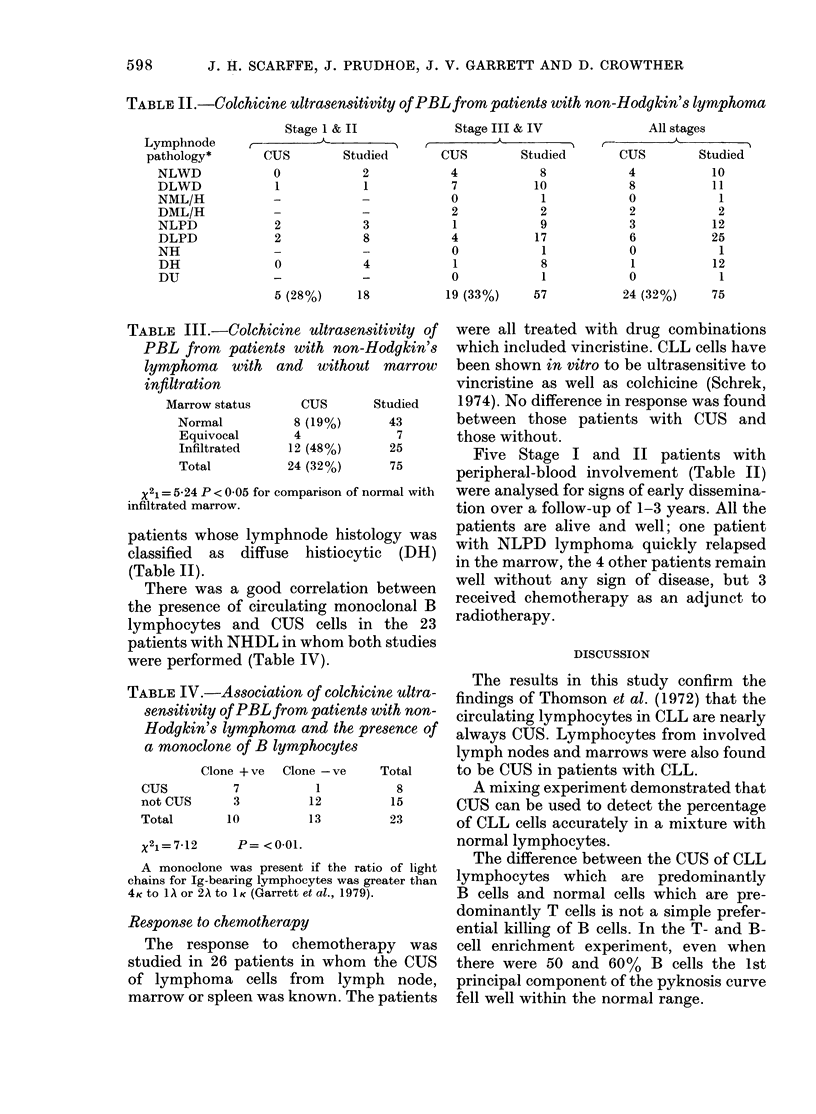

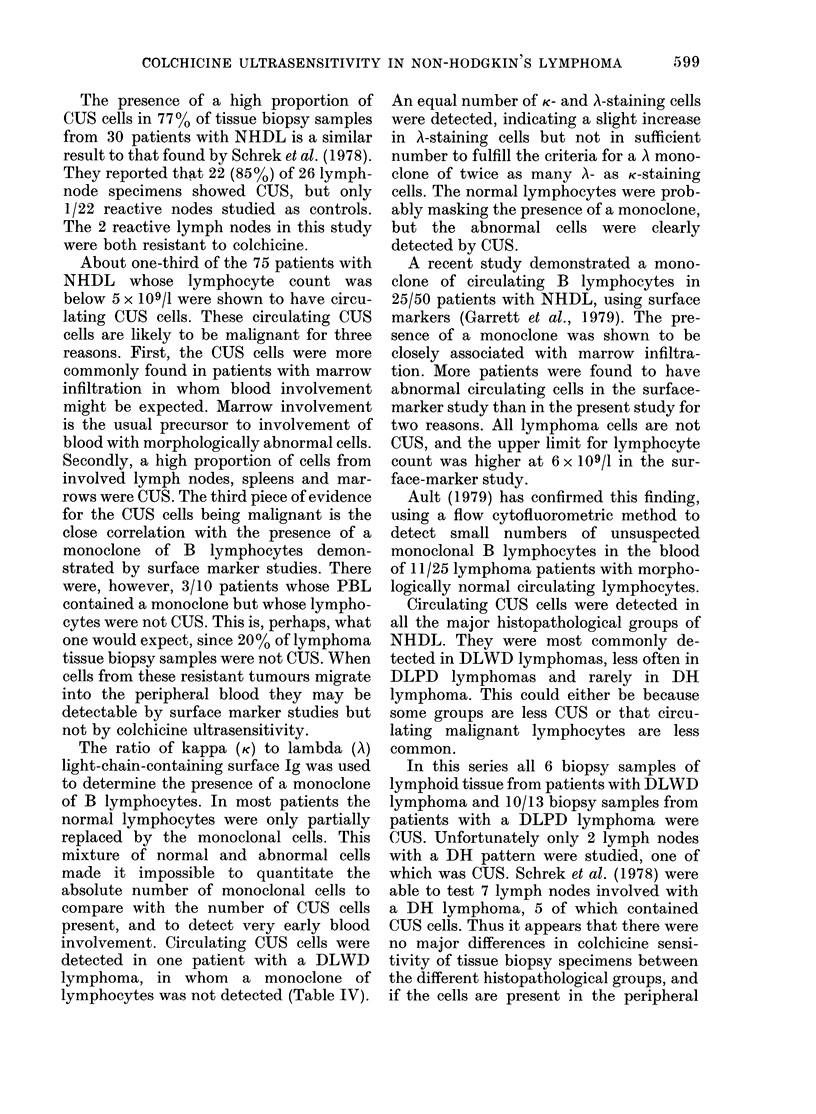

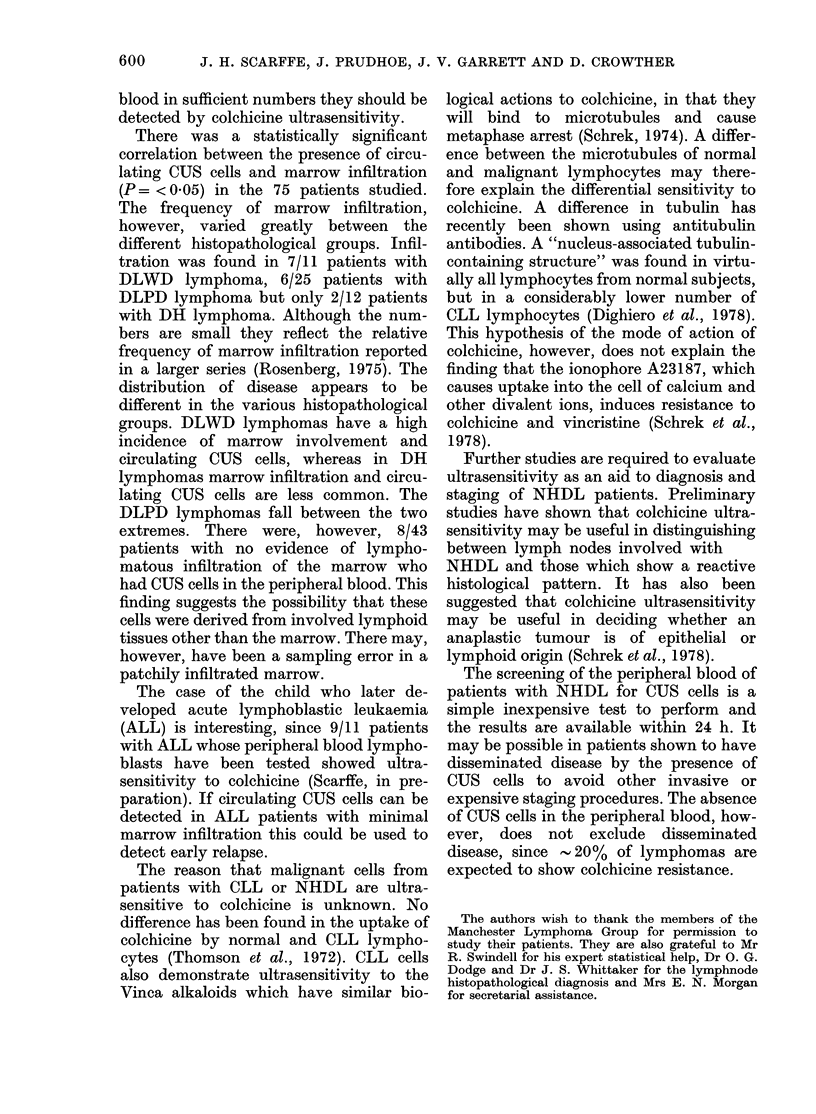

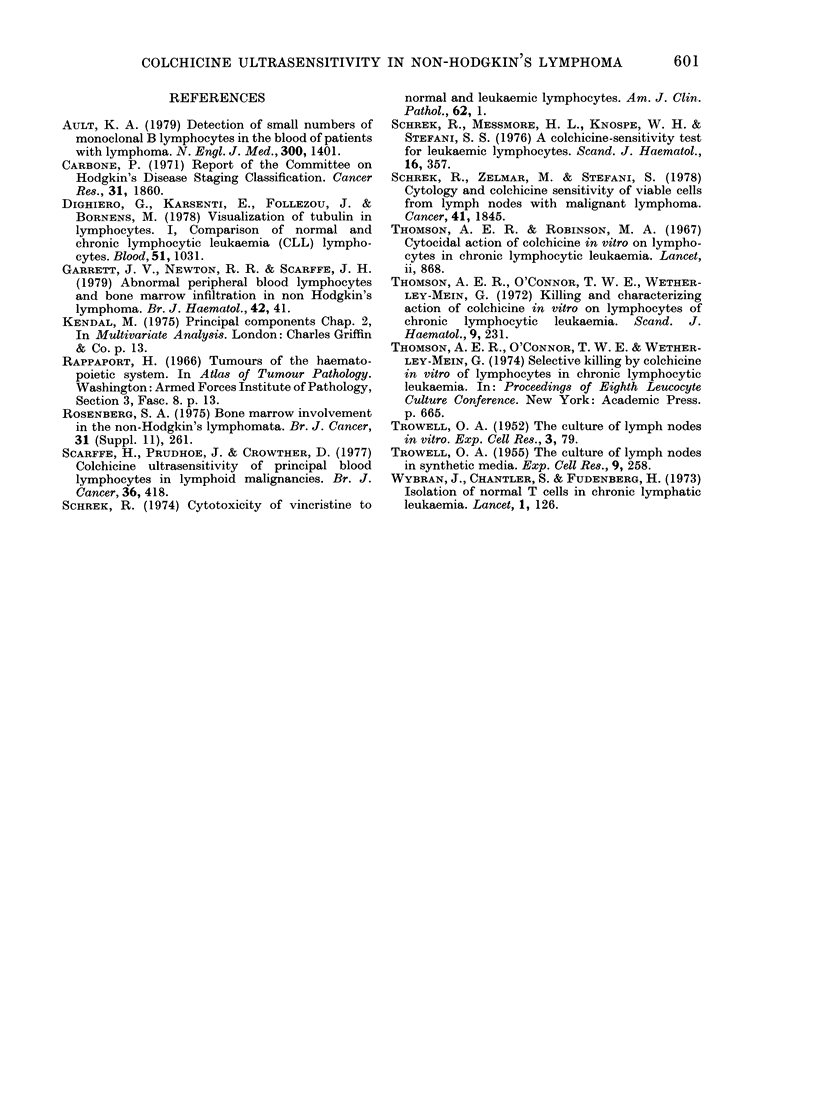


## References

[OCR_01034] Ault K. A. (1979). Detection of small numbers of monoclonal B lymphocytes in the blood of patients with lymphoma.. N Engl J Med.

[OCR_01039] Carbone P. P., Kaplan H. S., Musshoff K., Smithers D. W., Tubiana M. (1971). Report of the Committee on Hodgkin's Disease Staging Classification.. Cancer Res.

[OCR_01044] Dighiero G., Karsenti E., Follezou J. Y., Bornens M. (1978). Visualization of tubulin in lymphocytes. I. Comparison of normal and chronic lymphocytic leukemia (CLL) lymphocytes.. Blood.

[OCR_01051] Garrett J. V., Scarffe J. H., Newton R. K. (1979). Abnormal peripheral blood lymphocytes and bone marrow infiltration in non-Hodgkin's lymphoma.. Br J Haematol.

[OCR_01068] Rosenberg S. A. (1975). Bone marrow involvement in the non-Hodgkin's lymphomata.. Br J Cancer Suppl.

[OCR_01084] Schrek R., Messmore H. L., Knospe W. H., Stefani S. S. (1976). A colchicine-sensitivity test for leukaemic lymphocytes.. Scand J Haematol.

[OCR_01090] Schrek R., Molnar Z., Stefani S. S. (1978). Cytology and colchicine sensitivity of viable cells from lymph nodes with malignant lymphoma.. Cancer.

[OCR_01121] TROWELL O. A. (1955). The culture of lymph nodes in synthetic media.. Exp Cell Res.

[OCR_01104] Thomson A. E., O'Connor T. W., Wetherley-Mein G. (1972). Killing and characterizing action of colchicine in vitro on lymphocytes of chronic lymphocytic leukaemia.. Scand J Haematol.

[OCR_01096] Thomson A. E., Robinson M. A. (1967). Cytocidal action of colchicine in vitro on lymphocytes in chronic lymphocytic leukaemia.. Lancet.

[OCR_01125] Wybran J., Chantler S., Fudenberg H. H. (1973). Isolation of normal T cells in chronic lymphatic leukaemia.. Lancet.

